# Treatment of Puerperal Endometritis

**Published:** 1909-01

**Authors:** 


### ABSTRACTS.

### TREATMENT OF PUERPERAL ENDOMETRITIS.

Prof. E. C. Dudley writes in his Principles and Practice of Gynecology (Lea and Febiger, Philadelphia, 1908): The endometrium, after an irrigation with a 1:500 formalin solution or some other disinfectant, is swabbed out (using fresh gauze on the forceps), with a 10 per cent creolin solution or some other disinfectant, and then ½ oz. of Credé's ointment of argentum colloidale introduced. Thereafter the uterus is not invaded unless some special indications arise.

A dram of Credé's ointment may be rubbed in thoroughly for twenty minutes each day over the back or abdomen. Intravenous injection of 15 grains of a 2 per cent. solution of argentum colloidale, once a day, is a recent and promising therapeutic resource.

In the treatment of general infections of the peritoneum after operations, Credé's ointment of argentum colloidale, one dram a day thoroughly rubbed in for two to twenty minutes, and intravenous injection of a 2 per cent. solution of argentum colloidale once a day in doses of 15 grains, are among the strongly recommended measures.

